# Quality of information in gestational diabetes mellitus videos on TikTok: Cross-sectional study

**DOI:** 10.1371/journal.pone.0316242

**Published:** 2025-02-06

**Authors:** Genyan Jiang, Lei Chen, Lan Geng, Yuhan Zhang, Zhiqi Chen, Yaqi Zhu, Shuangshuang Ma, Mei Zhao

**Affiliations:** School of Nursing, Anhui Medical University, Hefei, China; Wenzhou-Kean University, CHINA

## Abstract

**Background:**

TikTok is an important channel for consumers to obtain and adopt health information. However, misinformation on TikTok could potentially impact public health. Currently, the quality of content related to GDM on TikTok has not been thoroughly reviewed.

**Objective:**

This study aims to explore the information quality of GDM videos on TikTok.

**Methods:**

A comprehensive cross-sectional study was conducted on TikTok videos related to GDM. The quality of the videos was assessed using three standardized evaluation tools: DISCERN, the Journal of the American Medical Association (JAMA) benchmarks, and the Global Quality Scale (GQS). The comprehensiveness of the content was evaluated through six questions covering definitions, signs/symptoms, risk factors, evaluation, management, and outcomes. Additionally, a correlational analysis was conducted between video quality and the characteristics of the uploaders and the videos themselves.

**Results:**

A total of 216 videos were included in the final analysis, with 162 uploaded by health professionals, 40 by general users, and the remaining videos contributed by individual science communicators, for-profit organizations, and news agencies. The average DISCERN, JAMA, and GQS scores for all videos were 48.87, 1.86, and 2.06, respectively. The videos uploaded by health professionals scored the highest in DISCERN, while the videos uploaded by individual science communicators scored significantly higher in JAMA and GQS than those from other sources. Correlation analysis between video quality and video features showed DISCERN scores, JAMA scores and GQS scores were positively correlated with video duration (*P*<0.001). Content scores were positively correlated with the number of comments (*P*<0.05), the number of shares (*P*<0.001), and video duration (*P*<0.001).

**Conclusion:**

We found that the quality of GDM video on TikTok is poor and lack of relevant information, highlighting the potential risks of using TikTok as a source of health information. Patients should pay attention to identifying health-related information on TikTok.

## Introduction

Gestational diabetes mellitus (GDM) is a specific type of diabetes that occurs during pregnancy. It is characterized by the first appearance of abnormal glucose tolerance during pregnancy in women who had normal glucose tolerance before pregnancy or had potential pre-existing impaired glucose metabolism [1]. Reports have indicated that GDM can increase the long-term risk of cardiovascular diseases, type 2 diabetes, and hypertension in pregnant women [2, 3]. According to extensive data analysis, the incidence of GDM in the United States can be as high as 18% in recent years, while in China, it has risen to 17.5%, posing a serious threat to the health of pregnant women. Many studies have shown that pregnant women generally lack knowledge about GDM [4–6]. Therefore, there is an urgent need to improve pregnant women’s awareness of GDM through effective publicity and health education.

With the development of mobile internet, short-video applications based on mobile platforms have rapidly emerged, becoming one of the mainstream ways to disseminate information [7]. TikTok, as one of the earliest platforms to launch the theme activity of "Healthy China", has gained widespread popularity with its unique algorithmic recommendations and personalized content delivery features [8]. The widespread dissemination of health information and the high viewership on TikTok have significantly improved patients’ awareness of diseases, influencing their healthcare-seeking behaviors and treatment outcomes [9]. However, the quality of disease-related videos on TikTok varies widely, making it challenging for patients to identify reliable information and potentially leading to the spread of misinformation [10–12]. We found that the quality of GDM-related content on TikTok has not been thoroughly reviewed. Therefore, this study aims to evaluate the quality of GDM-related videos on TikTok to provide accurate guidance for patients and content creators on the platform.

## Methods

### Search strategy

On December 15, 2023, we used three Chinese terms, "gestational diabetes mellitus", "pregnancy associated with diabetes", and "pregnancy hyperglycemia", to search for relevant GDM (gestational diabetes mellitus) videos on TikTok. In its search functionality, TikTok provides three methods for sorting the results: "overall ranking," "most recent," and "Most Liked." Overall ranking is the default sorting mode recommended by TikTok. Considering that most users tend to use the default value, we retrieved the top 100 videos in the overall ranking mode for each of the three keywords, resulting in a total of 300 videos. We chose a threshold number of 100 for two reasons. First, TikTok’s search functionality takes into account thematic relevance, with relevant GDM videos mostly appearing at the top of the results list. When results exceed 100, it becomes challenging to observe videos with higher relevance. Second, most ordinary health consumers apply the "minimum effort" principle in their online information-seeking activities. Therefore, they typically view the top search results instead of going very far [13].

**Fig 1:**
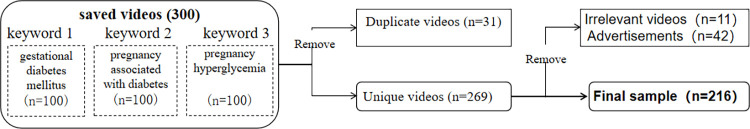
Search strategy and video screening procedure.

In order to select the most relevant videos, we excluded (1) duplicate videos (n = 31), (2) videos that were not directly related to the GDM theme (n = 11), and (3) advertisements (n = 42). Finally, a total of 216 videos were retained for data analysis (Fig 1). As the videos were publicly available, our collection and analysis complied with necessary terms and conditions.

#### Data extraction

We utilized Microsoft Excel to extract and encode basic information from each video. This included the video’s description, URL, upload date, duration (in seconds), uploader’s user ID, as well as the number of shares, likes, and comments (S1 Appendix). Based on the above mentioned information and home page certification, the TikTok authors were classified into 6 categories: (1) individual science communicators, (2) news agencies, (3) for-profit organizations, (4) health professionals, (5) nonprofit organizations, and, (6) general users.

#### Instruments

Videos were assessed from two aspects: the quality of information and their contents. Video quality was assessed using three rating scales. The first was the DISCERN tool [14] (S2 Appendix), which is a well-validated and widely used tool to help both consumers and healthcare professionals assess the quality of health-related content in videos. The second tool was the JAMA benchmark criteria (S3 Appendix) which use four standards to evaluate the quality of online information: authorship, attribution, disclosure, and currency [15]. For each standard, a score of 0 is given if it is not met and 1 if it is met, with a total score ranging from 0 to 4. The final tool was the Global Quality Score (GQS) (S4 Appendix), another widely used tool that evaluates the quality of health information in videos [12]. Researchers rate videos based on their reliability and content. Scores range from 1 to 5, with higher scores indicating better video quality and more comprehensive information [12].

Additionally, we adopted the six aspects proposed by Goobie et al. (definition of a disease, risk factors, evaluation, signs and symptoms, management, and outcomes) to assess video content [16]. Each aspect was rated on a scale of three items: not addressed (0 points), partially addressed (1 point), and fully addressed (2 points).

#### Coding procedure

All video content was independently rated and coded by two evaluators (GJ and LG), both of whom are nursing graduate students. One of them is a registered nutritionist. They both have experience in maternal and child nutrition management and health behavior change in gestational diabetes mellitus. The evaluation was conducted using DISCERN, JAMA benchmark criteria, GQS, and six questions proposed by Goobie et al. [16]. Before coding, a training session was conducted, during which the two raters individually scored and coded 50 videos. Any discrepancies arising from this process were discussed and resolved to ensure coding homogeneity. The coding process comprised three steps.

Firstly, we recorded basic information about the video publishers (e.g., account name, self-description, identity verification status) and video details (e.g., publication date, video length, likes, comments, shares). Regarding video publishers, we categorized video sources into two main types (i.e., individual users and organizational users) based on their account names and identity verification status. Additionally, we identified several subcategories within each source type based on account names, self-descriptions, and video publishing records. For example, if a video publisher described themselves as an "endocrinologist," we coded the source as a "Health professionals."

Subsequently, we used the six categories proposed by Goobie et al. to assess video content: definition of the disease, its signs and symptoms, risk factors, evaluation, management, and outcomes. After initial training, the two raters reached a consensus on whether a video contained content related to each category. Subsequently, one rater (GJ) performed the scoring for other videos, and the other rater (LG) verified the coding. During this process, any inconsistencies between the two raters were discussed and resolved.

In last step, we used DISCERN, JAMA benchmark criteria, and GQS to assess the quality of video information. Before scoring, both raters familiarized themselves with the instructions for DISCERN, JAMA, and GQS and discussed how to use these tools to assess video quality. The two raters independently scored all videos, and any discrepancies in the ratings were reconciled through collaborative discussion.

### Ethical considerations

This study did not involve clinical data, human specimens, or experimental animals. All information used in this study was sourced from publicly accessible TikTok videos, ensuring the protection of personal privacy. All our collection and analysis methods meet the terms and conditions of the platform. Additionally, this study did not involve any direct interaction with users, so no ethical review or trial registration was required.

### Statistical analysis

Data analysis was conducted using SPSS version 26.0. Multiple group comparisons of scores were performed using the Kruskal-Wallis test. Cohen’s kappa was employed to assess the inter-rater reliability in judgments or scores [17]. Quantitative data were presented as mean ± standard deviation, while qualitative data were expressed as counts (percentages). Spearman correlation analysis was used to evaluate the relationships between quantitative variables. A significance level of *P* < .05 was considered statistically significant.

## Results

### Video characteristics

GDM-related videos on TikTok were primarily provided by individual users (96.3%) and organizational users (3.7%). Among individual users, health professionals produced the majority of videos (75.0%), followed by general users (18.4%) and individual science communicators (2.7%). As for organizational users, videos were equally split between for-profit organizations and news agencies (1.8% each), with no videos from nonprofit organizations identified (Table 1).

**Table 1:** Characteristics of the sources of chronic obstructive pulmonary disease–related TikTok videos (N = 216).

Source	Description	Videos, n (%)
**Individual users**		
**General users**	Common TikTok users	40(18.5)
**Individual science communicators**	Individuals who are engaged in scientific communication (eg, popular science writers)	6(2.8)
**Health professionals**	Individuals who identify themselves as health professionals (eg, doctors and nurses)	162(75.0)
**Organizational users**		
**For-profit organizations**	Private sector organizations	4(1.8)
**Nonprofit organization**	Organizations or hospitals operating in the public sector	0(0)
**News agencies**	Organizations providing news services	4(1.8)

Note: The sum of percentages exceeding 100% in the table is due to rounding.

In our sample, video duration ranged from 5 seconds to 442 seconds. Videos from news agencies were the longest, followed by those from individual science communicators and general users. Videos from other sources averaged less than one minute in length (Table 2).

**Table 2:** Characteristics of chronic obstructive pulmonary disease–related TikTok videos, by source.

Source of videos	Days on TikTok, median (IQR)	Video duration (seconds), median (IQR)	Number of likes, median (IQR)	Number of comments, median (IQR)	Number of shares, median (IQR)	Number of collections, median (IQR)
**Individual users**						
**Health professionals**	433(262–598)	44(34–66)	487(150–2013)	188(23–634)	131(37–680)	72(22–308)
**Individual science communicators**	748(203–1039)	32(22–214)	22500(1052–46250)	3337(371–3799)	3075(144–9273)	2180(79–5335)
**General users**	434(254–626)	45(27–77)	1566(516–4058)	740(162–1544)	225(112–991)	176(54–518)
**Organizational users**						
**For-profit organizations**	310(119–801)	32(30–80)	988(155–3463)	264(26–500)	626(51–2083)	294(9–3463)
**Nonprofit organizations**	0(0)	0(0)	0(0)	0(0)	0(0)	0(0)
**News agencies**	561(186–797)	100(52–206)	244(37–791)	66(3–312)	126(8–388)	32(4–76)
**Overall**	435(258–615)	44(32–71)	613(184–2245)	230(30–819)	186(49–770)	94(23–391)

The most recently uploaded video was posted 1 day before data collection, while the oldest video on TikTok had been present for over 4 years. The number of likes per video ranged from 2 to 72,000, and the number of comments varied from 0 to 6,647. Except for for-profit organizations, videos uploaded by individual science communicators received the most likes and comments, while videos from news agencies received the fewest. Since their release, the entire sample of videos has been shared 260,623 times.

### Video content

**Fig 2:**
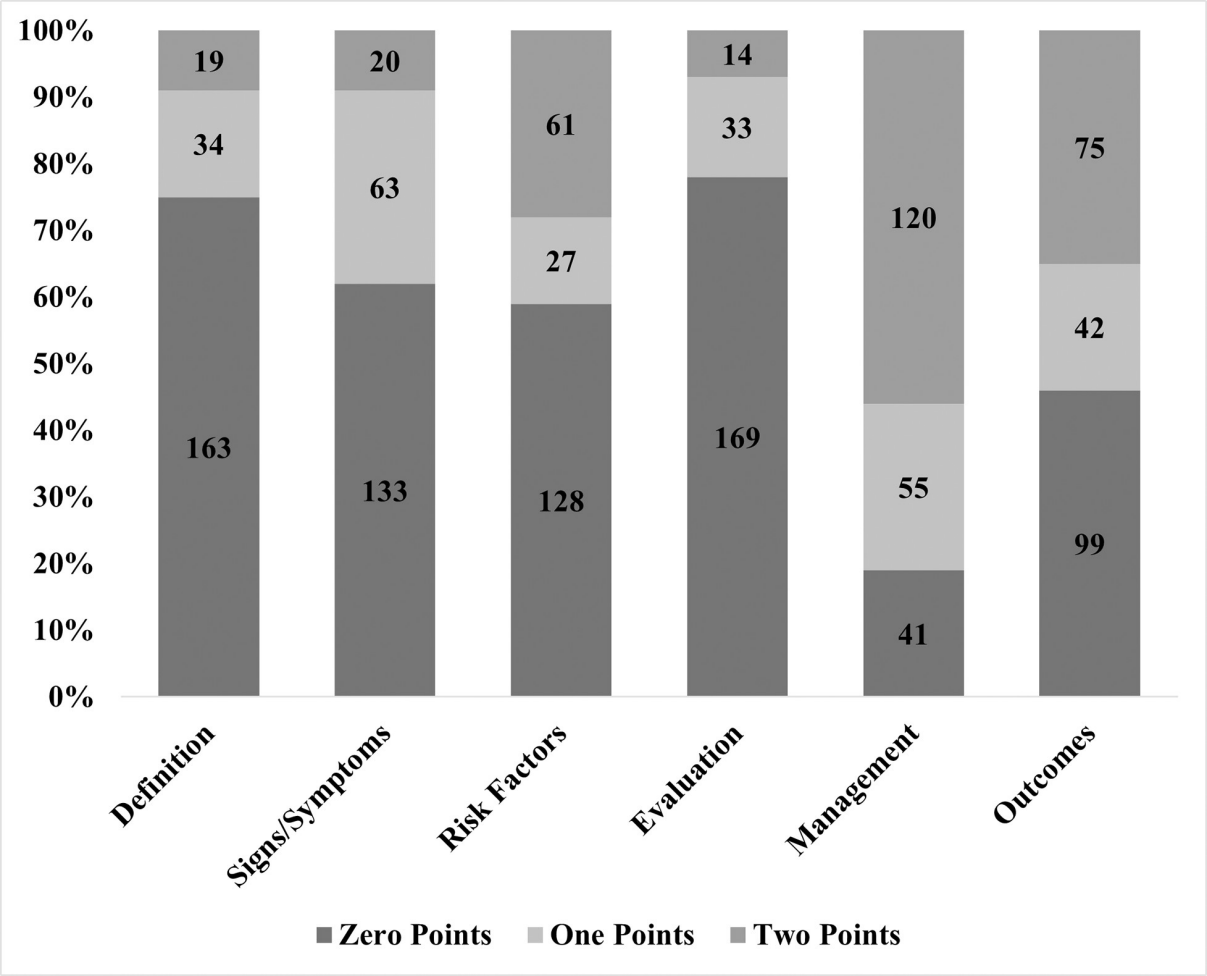
Percentage of videos addressing each gestational diabetes mellitus topic.

As shown in Fig 2, these videos covered the six predefined content areas to varying degrees. Of the 216 videos, 120 (55.6%) broadly discussed gestational diabetes mellitus (GDM) management, while 41 (19.0%) did not mention management at all. Nearly one-third (34.7%) of the videos explored GDM outcomes in detail, with 42 videos (19.4%) partially addressing outcomes. However, only a few videos covered the definition, signs/symptoms, and risk factors of GDM. The topic of GDM evaluation was the least discussed, with only 14 videos (6.5%) addressing it thoroughly, and 169 videos (78.2%) not mentioning it at all.

### Information quality

The average score given for the videos on the DISCERN instrument by both raters was 48.87 (out of 80), indicating that the overall quality of these videos was fair. (An average total score of 16–26 is very poor, a score of 27–38 is poor, a score of 39–50 is fair, a score of 51–62 is good, and a score >63 is excellent). The inter-rater reliability (Cohen’s κ) was 0.799 (P < .001), indicating a high level of agreement between the raters. Among videos produced by different types of creators, those made by health professionals had the highest average score (49.72), followed by individual science communicators (48.00) and news agencies (47.88).

We calculated the average scores for each DISCERN item in the overall sample. The scores ranged from 2.94 to 4.00 (average 3.05). The scores for the eight items assessing publication reliability (items 1–8) ranged from 1.88 to 4.13 (average 3.04). The scores for the seven items evaluating the quality of treatment choice information (items 9–15) ranged from 2.29 to 4.29 (average 3.12). The item measuring the overall information quality (item 16) obtained an average score of 2.56 out of 5.

We divided the DISCERN items into three parts based on the original instrument: publication reliability, quality of treatment choice information, and overall publication rating (Table 3). Videos published by health professionals had the highest reliability, while those provided by for-profit organizations had the lowest. Overall, there were significant differences across the videos from different sources in terms of publication reliability, quality of treatment choice information, overall publication rating, and DISCERN total scores (Table 3).

**Table 3:** Quality scores of videos by different types of uploaders (DISCERN, JAMA, and GQS).

Source of videos	Health professionals (n = 162)	General users (n = 40)	Individual science communicators (n = 6)	For-profit organizations (n = 4)	News agencies (n = 4)	*P* value^a^
**Reliability of the videos (items 1–8), mean (SD)**	24.73(3.28)	23.06(2.90)	23.33(3.01)	23.00(3.16)	23.13(2.53)	0.031
**Quality of treatment choices(items 9–15), mean (SD)**	22.18(3.09)	20.39(2.68)	22.33(4.32)	21.13(2.25)	22.00(4.55)	0.021
**Overall information quality (item 16), mean (SD)**	2.81(0.73)	2.42(0.68)	2.33(0.82)	2.50(0.58)	2.75(0.50)	0.045
**Total DISCERN scored, mean (SD)**	49.72(5.62)	45.87(5.13)	48.00(6.72)	46.63(5.22)	47.88(4.33)	0.004
**JAMA, mean (SD)**	1.91(0.41)	1.65(0.53)	2.17(0.75)	1.75(0.50)	1.50(0.58)	0.003
**GQS, mean (SD)**	2.37(0.56)	2.10(0.63)	2.67(1.03)	1.50(0.58)	2.25(0.50)	0.008

^a^*P* values were calculated with the Kruskal-Wallis H test.

The overall quality of each video was also assessed using the JAMA benchmark and GQS. Unfortunately, none of the videos in our study met all the JAMA standards. The average JAMA score was 1.86, with videos from individual science communicators scoring the highest (2.17) and news agencies scoring the lowest (1.50). The average GQS score was 2.06, falling below the GQS benchmark for high-quality videos (4 points). In the subgroup analysis, individual science communicators had the highest average GQS score (2.67), while for-profit organizations had the lowest (1.50) (Table 3).

### Correlation analysis

Spearman correlation analysis revealed positive correlations among the following variables: likes and comments (*r* = 0.901, *P*<0.001), likes and shares (*r* = 0.886, *P*<0.001), likes and video duration (*r* = 0.245, *P*<0.001), comments and shares (*r* = 0.861, P<0.001), comments and video duration (*r* = 0.169, *P*<0.05), and shares and video duration (*r* = 0.318, *P*<0.001) (Table 4).

**Table 4:** The relationship level between video variables.

Variable	Likes		Comments		Shares	
	*r*	*p*	*r*	*p*	*r*	*p*
**Likes**	-	-	0.901	<0.001	0.886	<0.001
**Comments**	0.901	<0.001	-	-	0.861	< .001
**Shares**	0.886	<0.001	0.861	<0.001	-	-
**Days since upload**	0.077	0.260	0.064	0.349	-0.009	0.900
**Video duration**	0.245	<0.001	0.169	<0.05	0.318	<0.001

DISCERN scores were positively correlated with video duration (*r* = 0.359, *P*<0.001). The JAMA benchmark scores were negatively correlated with the number of days since upload (*r* = -0.142, *P*<0.05) and positively correlated with video duration (*r* = 0.242, *P*<0.001). GQS scores were positively correlated with video duration (*r* = 0.268, *P*<0.001). Finally, Content scores were positively correlated with the number of comments (*r* = 0.147, *P*<0.05), the number of shares (*r* = 0.186, *P*<0.001), and video duration (*r* = 0.405, *P*<0.001) (Table 5).

**Table 5:** Correlations between between video quality scores and video variables.

Variable	DISCERN		JAMA		GQS		Content	
	*r*	*p*	*r*	*p*	*r*	*p*	*r*	*p*
**Likes**	-0.003	0.970	-0.131	0.054	0.030	0.658	0.133	0.051
**Comments**	-0.025	0.713	-0.086	0.208	0.030	0.663	0.147	<0.05
**Shares**	0.117	0.085	-0.014	0.841	0.051	0.452	0.186	<0.001
**Days since upload**	-0.092	0.179	-0.142	<0.05	-0.077	0.261	-0.036	0.599
**Video duration**	0.359	<0.001	0.242	<0.001	0.268	<0.001	0.405	<0.001

## Discussion

The appearance of health-related short videos on the TikTok platform marks a new trend of combining short videos with health education [18]. As a new and prolific medium, TikTok provides a large number of health-related videos [19]. However, the misinformation spread in some of these videos can also quickly proliferate and impact public health [20–22]. Therefore, we conducted this study to assess the quality and credibility of health information related to gestational diabetes mellitus (GDM) published on the TikTok platform.

In this study, we found that health professionals made up the majority of video uploaders, accounting for 162 (75.0%) videos, while the number of videos uploaded by the other five types of contributors was relatively small. Previous studies have shown that health professionals and organizations can effectively utilize social media for health education and public health advocacy [10]. However, our data indicated that organizational users promote GDM-related information on the TikTok platform less frequently than health professionals. Considering the busy schedules of healthcare personnel, we suggest that public hospitals or health organizations establish dedicated teams to produce and manage professional short videos, providing more high-quality content for patients.

Moreover, our findings revealed that the overall quality of TikTok’s GDM-related short videos is unsatisfactory. This finding is consistent with a number of evaluation results on the related video quality on the platform [12, 23–26]. We attribute this to the lack of stringent content review mechanisms on short video platforms, and the absence of restrictions based on the type of content creator, which may affect the content quality. Some previous studies have found that the quality of videos is not necessarily related to their popularity [23, 27]. Therefore, it is necessary for short video platforms to strengthen the supervision of health communication content and ensure the scientific validity of the information being disseminated.

Additionally, our study found that the content in these videos is often unbalanced. More than half of the videos did not cover topics such as definitions, signs/Symptoms, risk factors, and assessments. This may be due to the unique nature of short videos, making it challenging to comprehensively explain multiple aspects within a few minutes, resulting in lower completeness scores. However, the accuracy of the information covered in the videos was deemed satisfactory. Previous studies have shown that the comprehensiveness of video content positively impacts a video’s popularity [9], so we suggest that relevant videos should appropriately explain definitions, expand content, and update treatment information to improve the completeness of the knowledge presented and meet users’ needs for comprehensive health information.

### Limitations and future research

There are several limitations to this study. First, we only evaluated short videos published on TikTok, and the quality of information on other platforms still requires further research. Second, since search results on TikTok are dynamic over time, the results may vary depending on the search date and time. Third, video sampling only retrieved the top 100 videos per search, which may result in insufficient coverage. Additionally, this study only included Chinese-language short videos, and videos in other languages still need to be evaluated.

## Conclusion

We found that the quality of GDM video on TikTok is poor and lack of relevant information, highlighting the potential risks of using TikTok as a source of health information. Patients should pay attention to identifying health-related information on TikTok.

## Supporting information

S1 Appendix Original data set used for the current study. (XLSX)

S2 Appendix The DISCERN instrument questionnaire. (DOC)

S3 Appendix The Journal of American Medical Association (JAMA) scoring. (DOCX)

S4 Appendix Global Quality Score (GQS). (DOCX)
