# Expression of Concern: Quality of information in gestational diabetes mellitus videos on TikTok: Cross-sectional study

**DOI:** 10.1371/journal.pone.0340234

**Published:** 2026-01-05

**Authors:** The PLOS One Editors

After this article [1] was published, concerns were identified by the *PLOS One* Editors regarding the article’s peer review. The Editors have no evidence of any author involvement in the peer review issues.

In addition, the Editors are concerned about adherence of this article to the journal’s third publication criterion [2]. We regret that the issues were not addressed prior to the article’s publication.

In light of the cumulative issues, the *PLOS One* Editors issue this Expression of Concern. Readers are advised to interpret the article [1] with caution.

Additionally, information about the article’s funding incorrectly appears in the Acknowledgements section, and the Funding statement incorrectly states that no funding was received. The correct Funding statement is: This study was supported by the National Natural Science Foundation of China (Grant No. 72474001), the Major Foundation of Social Science and Humanity Anhui Province Department of Education (Grant No. SK2021ZD0030), Anhui Provincial Natural Science Foundation (Grant No. 2308085MH285), and School of Nursing, Anhui Medical University Graduate Student Seedling Cultivation Program (Grant No. Hlqm12024083).
