# Supplementary material for: Quality of information in gestational diabetes mellitus videos on TikTok: Cross-sectional study
Source: PLoS One. 2025 Feb 6;20(2):e0316242. doi: 10.1371/journal.pone.0316242 (PMC11801523; doi:10.1371/journal.pone.0316242)
Supplement: S3 Appendix — (DOCX) [file pone.0316242.s003.docx]

**S3 Appendix. The Journal of American Medical Association (JAMA) scoring.**

| **Criteria** | **Description** |
| --- | --- |
| Authorship 作者身份 | Authors and contributors, their affiliations, and relevant credentials should be provided.  应提供作者和贡献者、他们的隶属关系和相关的证书。 |
| Attribution | References and sources for all content should be listed clearly, and all relevant copyright information noted.  所有内容均应列出清晰的参考资料和来源，并注明所有相关的版权信息。 |
| Currency | Website ownership should be prominently and fully disclosed, as should any sponsorship, advertising, underwriting, commercial funding arrangements or support, or potential conflicts of interest.  网站所有权应显著和充分地披露，如任何赞助、广告、承销、商业资金安排或支持，或潜在的利益冲突。 |
| Disclosure | Dates that content was posted and updated should be indicated.  应该注明内容被发布和更新的日期。 |
