# Supplementary material for: Quality of information in gestational diabetes mellitus videos on TikTok: Cross-sectional study
Source: PLoS One. 2025 Feb 6;20(2):e0316242. doi: 10.1371/journal.pone.0316242 (PMC11801523; doi:10.1371/journal.pone.0316242)
Supplement: S4 Appendix — (DOCX) [file pone.0316242.s004.docx]

| **GQS Definition**  S4 Appendix. Global Quality Score (GQS). | **Score** |
| --- | --- |
| Poor quality, poor flow of the video, most information missing, not at all useful for patients  视频质量差，视频流量差，大部分信息缺失，对患者根本没有任何用处 | 1 |
| Generally poor quality and poor flow, some information listed but many important topics missing, of very limited use to patients  一般质量差，流量差，一些信息列出，但许多重要的主题缺失，对患者的使用非常有限 | 2 |
| Moderate quality, some important information is adequately discussed  质量适中，一些重要的信息被充分讨论 | 3 |
| Good quality good flow, most relevant information is covered, useful for patients  质量好，最相关信息覆盖，对患者有用 | 4 |
| Excellent quality and flow, very useful for patients  质量和流量都很好，对病人非常有用 | 5 |
